# Improved Aflatoxins and Fumonisins Forecasting Models for Maize (PREMA and PREFUM), Using Combined Mechanistic and Bayesian Network Modeling—Serbia as a Case Study

**DOI:** 10.3389/fmicb.2021.643604

**Published:** 2021-04-13

**Authors:** Ningjing Liu, Cheng Liu, Tatjana N. Dudaš, Marta Č. Loc, Ferenc F. Bagi, H. J. van der Fels-Klerx

**Affiliations:** ^1^Wageningen Food Safety Research, Wageningen, Netherlands; ^2^Faculty of Agriculture, University of Novi Sad, Novi Sad, Serbia

**Keywords:** mycotoxins, prediction, corn, weather, unbalanced data, validation, *Aspergillus*, *Fusarium*

## Abstract

Contamination of maize with aflatoxins and fumonisins is one of the major food safety concerns worldwide. Knowing the contamination in advance can help to reduce food safety risks and related health issues and economic losses. The current study aimed to develop forecasting models for the contamination of maize grown in Serbia with aflatoxins and fumonisins. An integrated modeling approach was used, linking mechanistic modeling with artificial intelligence, in particular Bayesian network (BN) modeling. Two of such combined models, i.e., the prediction model for aflatoxins (PREMA) and for fumonisins (PREFUM) in maize, were developed. Data used for developing PREMA were from 867 maize samples, collected in Serbia during the period from 2012 to 2018, of which 190 were also used for developing PREFUM. Both datasets were split randomly in a model training set and a model validation set. With corresponding geographical and meteorological data, the so-called risk indices for total aflatoxins and total fumonisins were calculated using existing mechanistic models. Subsequently, these risk indices were used as input variables for developing the BN models, together with the longitudes and latitudes of the sites at which the samples were collected and related weather data. PREMA and PREFUM were internally and externally validated, resulting in a prediction accuracy of PREMA of, respectively, 83 and 70%, and of PREFUM of 76% and 80%. The capability of PREMA and PREFUM for predicting aflatoxins and fumonisins contamination using data from the early maize growth stages only was explored as well, and promising results were obtained. The integrated approach combining two different modeling techniques, as developed in the current study, was able to overcome the obstacles of unbalanced data and deficiency of the datasets, which are often seen in historical observational data from the food safety domain. The models provide predictions for mycotoxin contamination at the field level; this information can assist stakeholders of the maize supply chain, including farmers, buyers/collectors, and food safety authorities, to take timely decisions for improved mycotoxin control. The developed models can be further validated by applying them into practice, and they can be extended to other European maize growing areas.

## Introduction

Maize (*Zea mays*) is one of the main sources for food and feed production in the world (Chulze, 2010). In 2017, more than 197 million hectares were grown with maize worldwide resulting in production yield of 1.13 billion tons of maize (FAOSTAT, 2020). Ensuring the quality and safety of maize for feed and food production is essential. One of the major quality and safety concerns is infection of the maize plants with fungi and the contamination of maize kernels with mycotoxins, which are toxic secondary metabolite compounds of certain fungal species. In temperate and semi-tropical areas, which are the main maize growing areas in Europe, maize is vulnerable to the infection of *Aspergillus* spp. and *Fusarium* spp., mainly *Aspergillus flavus* and *Fusarium verticillioides* (Chulze, 2010). It has been shown that aflatoxins and fumonisins, which are of the associated mycotoxins of these fungi, have negative impacts on the health of human and animals (Zain, 2011). In human and animals, these mycotoxins have the potential to promote the formation of cancer. Furthermore, toxic metabolic compounds (mainly aflatoxin M_1_) can be found in the excreted milk of milk-producing animals, such as dairy cows and goats, after intake of mycotoxin-contaminated feed (Santos Pereira et al., 2019). Knowing mycotoxin contamination of maize at harvest already during the growing stage or close to harvest allows maize supply chain stakeholders, e.g., farmers, collectors, or feed producers, to take timely management actions so to prevent from safety issues in derived feed and food. Predictions of mycotoxins in maize can be used for decisions on keeping batches from particular fields separately, for routing and processing in the chain and/or for risk-based inspection. In the latter case, only the maize grown in those areas with estimated high probability to be contaminated with mycotoxins can be sampled for mycotoxin analyses. Such risk-based sampling and analyses procedures, focusing on areas or batches with a predicted high contamination and not collecting samples when the contamination is predicted to be low, will reduce monitoring costs. These needs of stakeholders of cereal supply chains to predict the contamination of mycotoxins in advance has stimulated the development of mathematical forecasting models.

In previous studies, several obstacles in achieving a satisfactory performance of mycotoxin forecasting models have been identified. One of the major obstacles is related to mycotoxin monitoring data often being unbalanced. Historical datasets with data collected from practice, needed for model development, often consist for the majority of the samples with low mycotoxin concentrations. Using these data as the training set for model development will result in bias of the forecasting model, especially when using empirical modeling (Cnaan et al., 1997).

Liu et al. (2018) performed a comparison between the empirical modeling method and the mechanistic modeling method using mycotoxin contamination data related to wheat. Their results showed that the empirical model resulted in lower performance in predicting samples with high mycotoxin levels than the mechanistic model. Similar results were obtained by Battilani et al. (2008), who reported a poor prediction performance of their model for the high contaminated samples. Even though the model explains 60% of the variability of mycotoxin contamination, validation results of using independent data suggested that contamination levels of 56% of samples were overestimated. However, estimating those highly contaminated samples correctly is critical to control the safety of the products. Compared with empirical models, mechanistic models simulate the biological processes of fungal development, interactions with plants, and the formation of mycotoxins. Knowledge from the biological domain helps to decrease the impact of artificial bias of the model. Furthermore, mechanistic models are less dependent on data and are more suitable for handling anomaly scenarios, such as climate change, as compared to empirical models. In addition to unbalanced datasets, another obstacle for increasing the performance of predictive models is missing data. Since forecasting model for mycotoxins need to take many variables into account, ensuring the availability of input data on all the model variables is often difficult. In some circumstances, a prediction has to be made before all input data values are available. This limits the use of empirical forecasting models.

Recently, with the introduction of machine learning algorithms, these two main obstacles can be overcome. Bayesian network (BN) modeling, one of the widespread machine learning model techniques, can very well deal with both unbalanced data and missing data (Wang and Yuan, 2004). BN models are developed using observational data. Such models make predictions by calculating conditional probabilities among the available variables in the dataset (Liu et al., 2018).

The aim of this study was to develop a modeling approach that combines mechanistic modeling and BN modeling for the prediction of aflatoxin and fumonisin contamination in maize. As a case study, such a combined modeling approach was developed for prediction of these toxins in maize grown in Serbia.

## Materials and Methods

### Data Collection

For the aims of this study, we used results of analyzing maize samples for concentrations of aflatoxins and fumonisins. Data related to 867 maize samples were collected in the years 2012–2018 from Serbia and analyzed for the concentration of total aflatoxins (AF). Of this total, 190 samples collected in the years 2016–2018 were also analyzed for total fumonisins (FU). All samples were collected from maize kernels harvested from a particular maize field of a particular arable farm. Geographic coordinates of the fields from which samples were collected had been recorded. Daily data of temperature (T, ^o^C), relative humidity (RH,%), and precipitation (R, mm) were acquired from the JRC European meteorological database^1^. According to the resolution of the meteorological database, the sample collection region was covered by a grid of squares (25 km × 25 km). The meteorological data in each square were simulated by the data recorded from the nearby automatic weather stations. Using the farm location, meteorological data of each of the collected sample were linked accordingly.

### Mycotoxins Analyses

Samples were collected during the maize harvest in the northern Serbian province of Vojvodina. Collection of samples was performed according to EU requirements (EC 401/2006) to account for irregular mycotoxin distribution in harvested maize. If the yield of the field was ≤10 tons, a total of 40 incremental samples were collected, with a weight of 100 g each, resulting into a 4-kg aggregate sample. If the yield on the field was 10–20 tons, a total of 60 incremental samples were collected, with a weight per incremental sample of 100 g, resulting in a 6-kg aggregate sample. Samples were immediately transported to the chemical laboratory where they were kept in a freezer at −20°C until chemical analysis. Before sample preparation and analysis, the samples were allowed to reach room temperature. Samples were prepared by milling on a laboratory mill until >93% passed through a sieve with a pore diameter of 0.8 mm. Five grams of each milled sample were used for the extraction with a 20-ml extraction solvent [acetonitrile–water–acetic acid (VWR, Vienna, Austria), 79:20:1, v/v/v] followed by a 1 + 1 dilution using acetonitrile–water–acetic acid (VWR, Vienna, Austria) (20:79:1, v/v/v) and an injection of 5 μl of diluted extract. Liquid chromatography–tandem mass spectrometry (LC-MS/MS) screening of target mycotoxins was performed at the Institute of Bioanalytics and Agro-Metabolomics, Department of Agrobiotechnology (IFA-Tulln), University of Natural Resources and Life Sciences, Vienna, with a QTrap 5500 LC-MS/MS System (Applied Biosystems, Foster City, CA, United States) equipped with a TurboIon Spray electrospray ionization (ESI) source and a 1290 Series HPLC System (Agilent, Waldbronn, Germany). Gemini^®^ C18-column, 150 mm × 4.6 mm i.d., 5 μm particle size, equipped with a C18 4 mm × 3 mm i.d. security guard cartridge (all from Phenomenex, Torrance, CA, United States) was used for chromatographic separation at 25°C. Chromatographic method and chromatographic and mass spectrometric parameters, together with method validation data, are described by Malachova et al. (2014). Electrospray ionization–tandem mass spectrometry (ESI-MS/MS) was performed in the time-scheduled multiple reaction monitoring (MRM) mode in both positive and negative polarities in two separate chromatographic runs per sample by scanning two fragmentation reactions per analyte. The MRM detection window was set to its expected retention time of ± 27 s and ± 48 s in the positive and the negative mode, respectively, for each analyte. Confirmation of positive identification was obtained by the acquisition of two MRMs per analyte. Additionally, the LC retention time and the intensity ratio of the two MRM transitions corresponded with the related values of an authentic standard within 0.1 min and 30% rel., respectively. Quantification was based on an external calibration with a serial dilution of a multianalyte stock solution, and results were corrected for apparent recoveries. The accuracy of the method is continuously verified by regular participation in proficiency testing schemes (Malachova et al., 2014; Malachova et al., 2015) organized by BIPEA (Gennevilliers, France).

### Model Development

#### Data Processing

Based on the mycotoxin concentration (either AFs or FUs), each analytical sample result was labeled as “low” or “high.” The applied thresholds for AFs and FUs were 10 μg/kg and 1,000 μg/kg, respectively, based on Commission Regulation (EC) No 401/2006 and Commission Regulation (EC) No 1881/2006. Nineteen out of the 867 samples analyzed for AFs and 7 out of the 190 samples analyzed for FUs were close to their respective thresholds.

Using the mechanistic models presented by, respectively, Maiorano et al. (2009) and Battilani et al. (2013), the aflatoxin risk index (ARI) and the fumonisin risk index (FRI) of the sample records were calculated for each day of the particular maize growing season (from flowering date to maturation date), in which the sample had been collected. The flowering date and maturation date were estimated using the sum of the daily average temperatures in the fields (Maiorano et al., 2009). Details of the calculations can be found in the two original cited papers. In order to run the mechanistic models in the different maize growth stages, the entire cultivation period from the maize flowering date to the maturation date was divided evenly, per sample record, into eight sub-periods (P1 to P8). Thus, the number of days in each sub-period may differ per sample record. In each sub-period, the sum of daily ARI and the sum of daily FRI were calculated. These total ARI and FRI for each of the eight sub-periods (ARI_P1, ARI_P2, … ARI_P8, and FRI_P1, FRI_P2, …, FRI_P8) were used as inputs for BN model development.

#### Model Development and Validation

The prediction model for aflatoxins in maize (PREMA) was built using a BN algorithm, Tree-Augmented Naive Bayes (Friedman et al., 1997). The dataset related to AFs, collected in the period 2012–2016 in Serbia, was split into a training set and an internal validation set, by randomly selecting 80 and 20% of the data, respectively. The input variables used in PREMA included latitude, longitude, and the ARIs in each of the eight sub-periods of each sample (ARI_P1 to ARI_P8). The AF forecasting model was developed by using the training set and then internally validated by using the internal validation set. An independent validation dataset was used for external validation of PREMA. This dataset consisted of the AF data collected in 2017 and 2018.

Similarly, the prediction model for fumonisins in maize (PREFUM) was trained by Tree-Augmented Naive Bayes, using the latitude, longitude, and the FRIs in each of the eight sub-periods per record (FIR_P1 to FRI_P8) as inputs. The FU data collected in 2016 and 2017 were randomly split into a training set and an internal validation set by using the ratio of 80/20. PREFUM was developed using the training set, and internally validated using the internal validation set. Then, PREFUM was externally validated using the FU data collected in 2018.

The performances of PREMA and PREFUM were evaluated by the using the following criteria: accuracy (percentages of samples being correctly classified as high or low contaminated), specificity (percentages of the high class samples being correctly classified), and sensitivity (percentages of the low class samples being correctly classified) (Kuhn and Johnson, 2013). The lower the specificity, the higher the percentage of false negatives would be.

#### Early Warning Test

The early warning performance of PREMA and PREFUM was tested using the two external validation datasets. The early warning performance is defined here as the ability of the model to provide correct predictions—early in the maize growing season—of mycotoxin contamination at harvest. PREMA and PREFUM were validated with the coordinates and respective risk indices in P1 (ARI and FRI). The related model accuracy, specificity, and sensitivity were calculated. Additionally, the area under the ROC curve (AUC) was determined, indicating the performance of the model at all classification levels. Subsequently, the models were run in a similar way for periods P1 and P2; the ARI and FRI indices in P1 and P2 were estimated, and the four abovementioned performance criteria (accuracy, specificity, sensitivity, and AUC) were calculated again. This was repeated, each time adding the next sub-period, until the ARI and FRI risk indices of all eight periods were involved.

Model development and risk index calculations were performed in R (version 3.5.0).

## Results

### Aflatoxins Model

**FIGURE 1:**
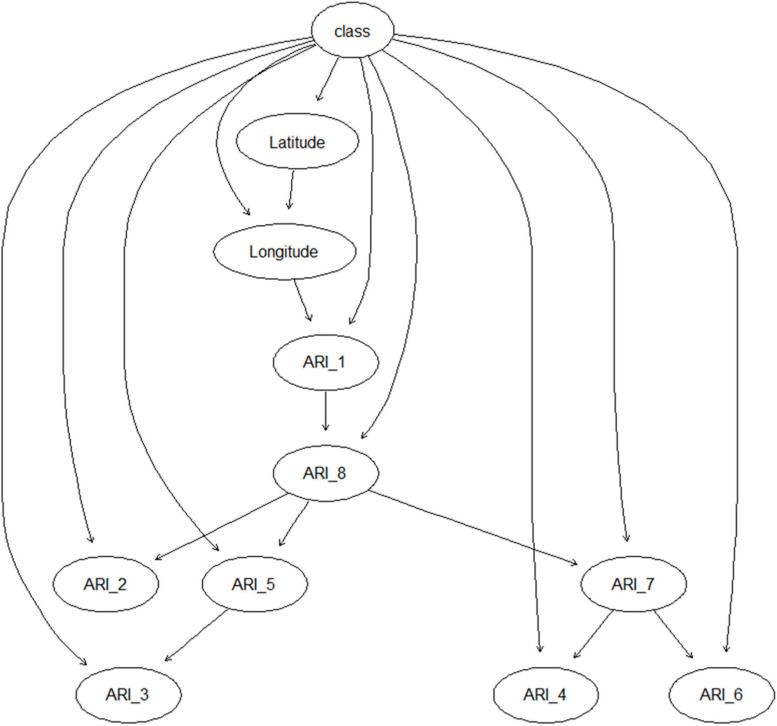
Structure of PREMA resulting from learning the model with field data collected in the years 2012–2016 in Serbia. Circles represent nodes of the Bayesian network model, and arrows indicate the relationship/conditional dependencies among the nodes. ARI_1–8: aflatoxins risk index in sub-periods 1–8.

Figure 1 present the structure of the PREMA resulting from training the model using the AF training set. Besides longitude and latitude, the risk indices in P1 and P8 were the ancestors of the risk indices in other periods (Figure 1), suggesting that the risk indices in these two periods directly impact the total AF contamination of maize. In Figure 1, below the node “ARI_P8,” there were three sub-groups: ARI in P2 (ARI_2), ARI in P5 and P3 (ARI_3 and ARI_5), and ARI in P4, 6, and 7 (ARI_4, ARI_6 and ARI_7). All the model variables (latitude, longitude, and the ARIs in the eight sub-periods) were linked with the total AF contamination class (low, high) in maize. Based on this BN model structure, the PREMA model was further developed and validated. The prediction results of using the training dataset, the internal validation set, and the external validation set are shown in Table 1. Using the model training set, performance results showed that 390 out of the 462 low-class records were correctly predicted as low class (specificity 84%), 116 out of 139 high-class records were correctly predicted as high class (sensitivity 84%), and, in total, 506 out of 601 records were correctly classified (overall accuracy 84%). Using the internal validation set, 104 of the 124 low-class records were correctly predicted as low (sensitivity 84%), 22 of 27 high-class records were correctly predicted as high (specificity 82%), and, in total, 126 of 151 records were correctly classified (overall accuracy 83%). External model validation results showed that 65 of the 97 low-class records were correctly classified as low class (sensitivity 67%), 17 of 20 high-class records were correctly classified as high class (specificity 85%) and, in total, 82 of 117 records were correctly classified (overall accuracy 70%).

**TABLE 1:** Performance of PREMA, comparing predicted total aflatoxin classes versus observed total aflatoxin classes.

		**Prediction^a^**								
		**Training set**			**Internal validation set**			**External validation set**		
		**High**	**Low**	**Accuracy**	**High**	**Low**	**Accuracy**	**High**	**Low**	**Accuracy**
Observation^b^	High	116	23	84%	22	5	83%	17	3	70%
	Low	72	390		20	104		32	65	

*^*a*^The aflatoxin contamination levels of samples from different datasets predicted by the model. ^*b*^The measured aflatoxin contamination levels of samples from different datasets.*

The performance of PREMA for early warning purposes was investigated as well. Table 2 presents the prediction results of using risk indices in the different maize growth stages. As can be seen from Table 2, when involving more and later maize sub-growth periods in the model, the overall accuracy of the model remains relatively constant, at around 70%. However, when the later growth periods are added, the specificity of the model predictions greatly increases, at the premise of only a small decrease in the sensitivity of the predictions. Since we especially aim to predict the highly contaminated maize fields correctly, we consider this an improvement of the overall model performance with the developing maize cultivation season.

**TABLE 2:** Performance of PREMA when using risk indices from different maize growth periods.

**Involved risk indices^a^**	**Accuracy (%)**	**Specificity (%)**	**Sensitivity (%)**	**AUC^b^**
P1	73	0	88	0.547
P1 to P2	74	45	79	0.584
P1 to P3	70	0	85	0.415
P1 to P4	59	0	71	0.503
P1 to P5	64	50	64	0.588
P1 to P6	60	50	62	0.579
P1 to P7	72	50	76	0.679
P1 to P8	70	85	67	0.859

*^*a*^Risk indices used in the BN model. ^*b*^Area under curve.*

With the progress of the growth season of maize, more ARIs can be involved in the AF prediction. When only the risk indices in the early growth stages are involved, the sensitivity of the prediction was relatively high, but the specificity was very low, suggesting a high percentage of false negatives. In this case, maize fields that are highly contaminated with AF could be predicted as low contaminated (negative), but fields that are not highly contaminated are predicted correctly. In other words, the model was partly biased, focusing more on maize with low contaminated levels rather than on highly contaminated maize. When the risk indices of the later growth stages were involved, the specificity of the prediction greatly increased to 85%, implying that the high contaminated fields are correctly predicted as being high contaminated. The discriminatory capacity of high class AF samples was improved. Compared with the model performance in the early maize growth stages, the model in the late growth stages provides relatively the same overall accuracy, but with higher specificity. Such improvement was significant since classifying the highly contaminated maize fields correctly is essential in the practical situation. Meanwhile, the AUC became larger, especially when the risk indices of P8 were used in the input dataset. Hence, with the involvement of ARIs in the late maize growth stages, close to full maturation, the forecasting model became more balanced for both high contaminated and low contaminated samples.

### Fumonisins Model

**FIGURE 2:**
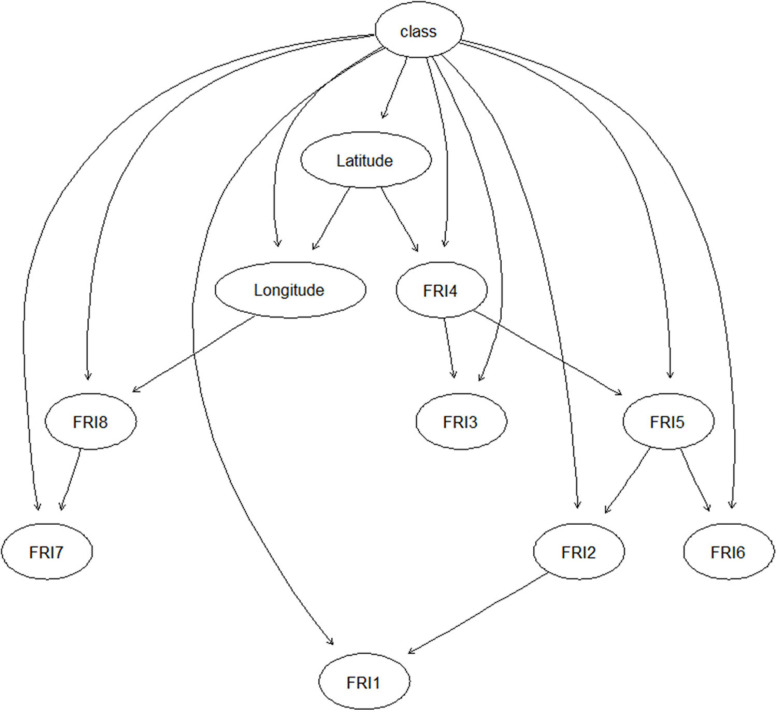
Structure of PREFUM resulting from learning the model with field data collected in the years 2016–2017 in Serbia. Circles represent nodes of the Bayesian network model, and arrows indicate the relationship/conditional dependencies among the nodes. FRI_1–8: fumonisins risk index in time sub-periods 1–8.

The structure of PREFUM is presented in Figure 2. Latitude was directly/indirectly connected with the FRIs in all eight sub-periods and thus had an effect on the FU contamination class (low, high) in each sub-period of maize growing, while longitude was only linked with the FRIs in P7 and P8. The FRI in these two sub-periods had no connection with the FRI in the earlier periods.

Table 3 shows the performance PREFUM for predicting total fumonisins in maize, according to the structure presented in Figure 2, using the three different types of model testing and validation. Using the model training set, the model sensitivity, specificity, and accuracy were 74% (51 of 69), 72% (28 of 39), and 73% (79 of 108), respectively. Using the internal model validation set, the model sensitivity, specificity, and accuracy were, respectively, 86% (18 of 21), 63% (10 of 16), and 76% (28 of 37). The model was further validated by an external validation set, using results of samples that had been collected in a different year. In this case, the model sensitivity, specificity, and accuracy were 86% (32 of 36), 50% (4 of 8), and 80% (36 of 45).

**TABLE 3:** Performance of PREFUM, comparing predicted total fumonisin classes versus observed total fumonisin classes.

		**Prediction^a^**								
		**Training set**			**Internal validation set**			**External validation set**		
		**High**	**Low**	**Accuracy**	**High**	**Low**	**Accuracy**	**High**	**Low**	**Accuracy**
Observation^b^	High	28	11	73%	10	6	76%	4	4	80%
	Low	18	51		3	18		5	32	

*^*a*^The fumonisin contamination levels of samples from different datasets predicted by the model. ^*b*^The measured fumonisin contamination levels of samples from different datasets.*

Similar to PREMA, the ability of PREFUM for early warning was explored as well. Table 4 presents the prediction performance of PREFUM when FRIs in different maize growth periods (P1–P8) were included, each time adding an additional sub-period. The total accuracy of the model prediction increased when FRIs in the late growth periods were also involved. Also, the model’s AUC was much higher when the later maize growth periods were involved as compared to the early growth periods, especially after FRI in P4 was involved in the model.

**TABLE 4:** Performance of fumonisins model when using risk indices from different maize growth periods.

**Involved risk indices in sub-period P^a^**	**Accuracy (%)**	**Specificity (%)**	**Sensitivity (%)**	**AUC^b^**
P1	49	51	100	0.57
P1 to P2	49	51	90	0.5
P1 to P3	51	40	90	0.51
P1 to P4	76	78	63	0.79
P1 to P5	76	86	63	0.79
P1 to P6	80	86	50	0.78
P1 to P7	80	86	50	0.73
P1 to P8	80	86	50	0.78

*^*a*^Risk indices used in the BN model. ^*b*^Area under curve.*

## Discussion

To date, several studies focused on developing forecasting models for mycotoxins in grains, mainly focusing on deoxynivalenol (DON) in wheat (Van der Fels-Klerx et al., 2010) and aflatoxins in maize (Battilani et al., 2013), using empirical and mechanistic modeling approaches. Only recently has the use of BN modeling for the aims of forecasting mycotoxins been explored by Liu et al. (2018). These authors used BN modeling for the prediction of DON in wheat in the Netherlands and compared this machine learning technique with both the empirical and mechanistic approach, by developing the three types of models using the same dataset. Results of their model comparison showed that BN modeling outperformed the empirical and mechanistic models for the case of predicting DON in wheat in the country. The current study is the first one that combines the two modeling approaches of mechanistic and BN modeling, by using the estimated risk index of the mechanistic model as input for training the BN model structure. This approach is especially powerful when the available dataset is unbalanced, which is often the case with using mycotoxin monitoring data.

The learned BN model structure was different for PREMA and PREFUM, as can be seen from Figures 1, 2. Different model structures suggest different relationships between the variables in each model. In PREMA, latitude and longitude were important for the ARI in all eight sub-periods of maize cultivation, while in PREFUM, only latitude was important for the eight FRIs. The underlying reason can be sought in the distribution of the responsible species for aflatoxins (*A. flavus*) and fumonisins (*Fusarium* spp.) in Serbia, and their distribution in the country. Fusarium species require lower temperatures for growth and mycotoxin production than *A. flavus*, and mycotoxins from Fusarium species have traditionally been associated with temperate climate regions, whereas *A. flavus* is seen in warm climate regions (Bandyopadhyay et al., 2016). Fusarium species are commonly present in maize grown in Serbia, especially in years with high precipitation and low temperatures (Jajić et al., 2008). Lević et al. (2012) state that the incidences of *F. graminearum*, *F. oxysporum*, *F. subglutinans*, and *F. verticillioides* have changed over the last years: the incidence of *F. subglutinans* has reduced, while the incidence of *F. verticillioides*, a fumonisin producer, has increased. It can be expected that the incidence of *F. verticillioides* will further increase in the future due to global warming, as this Fusarium species is more frequently seen in years with higher temperatures (Lević et al., 2004). The natural occurrence of *A. flavus* infection of maize is not very common under Serbia’s typical climatic conditions; however, epidemic outbreaks can happen in years with extreme weather conditions (high temperatures and prolonged drought) such as in 2012 and 2015 (Kos et al., 2013; Janić Hajnal et al., 2017; Savić et al., 2020).

In PREMA, ARIs in P1 and P8 were the ancestors of the ARIs in other periods, illustrating the contributions of these two AF risk indices to the model. These results are consistent with the results of the early warning testing of the model as presented in Table 2. From this table, it can be seen that the AUC significantly increased after ARI in P8 was included as input (one-sample *t* test, *P* < 0.05). Compared with the other three model performance criteria used, i.e., accuracy, sensitivity, and specificity, the AUC reflects the model discriminating capacity in a more comprehensive way, taking into account the impacts of data distribution and the values of classification boundary. The closer the AUC to 1, the better the model discrimination capacity. Sub-period 8 is the last period before the maize harvest, and apparently in Serbia, a great part of the aflatoxins are formed just before the maize harvest. According to the results of Payne and Widstrom (1992), rain prior to or during the harvest has severe impact on the contamination of aflatoxins in maize. Similarly, the FRI in P4 was a major contributor for explaining total fumonisin contamination in PREFUM (Figure 2 and Table 4). The performance of PREFUM significantly increased after involving the FRI in P4, which is the time period in between maize flowering and full maturation.

In previous research, several forecasting models for mycotoxins in maize have been developed with varying performances. Battilani et al. (2008) developed a logistic regression (LR) model to predict fumonisin contamination in maize. The model explained 60% of the total variability, with 58% of the samples correctly classified, among which 41% were correctly classified high contaminated samples. Later, Battilani et al. (2013) developed a mechanistic model to calculate ARI in maize, which was followed by a LR model to predict aflatoxins contamination in maize based on the estimated ARI. Their model performance showed that 15 out of 33 (45%) and 3 out of 22 (14%) contaminated records using, respectively, the training set and validation set could be correctly predicted as positive.

In the current study, with the help of BN modeling, the ability to correctly predict contaminated and non-contaminated field samples was higher than in the previous studies that used LR modeling. This may be related to the differences in characteristics of the BN model and the LR model. Compared with BN model, the LR model is based on a list of restricted statistical assumptions, two of which are linearity in the logit and additivity of model input values (Menard, 2002). Therefore, complex transformations (e.g., square root transformation, log transformation, and cox–cox transformation) of the input data are needed before the input dataset meets these assumptions. These complex transformations and investigation of the logit-linear relationships between the dependent variables and independent model variables can be hard to conduct. In the case of LR modeling for mycotoxin predictions, such assumptions cannot be met all the time, which will lead to instability of the model. On the contrary, BN models can be developed without the assumptions of linearity in the logit and/or additivity. Such intrinsic advantages make the BN model more robust in case pre-knowledge of mycotoxin contamination is limited. Furthermore, LR modeling cannot deal very well with interactions of the independent variables in large datasets (Lee et al., 2005). The number of possible interactions between variables exponentially increases with more variables in the dataset, which makes the model specification complex and difficult. Hence, in the LR model, only a restricted number of variables and interactions can be included. The BN model investigates the relationships between the variables (Friedman et al., 1997), which helps in dealing with a larger set of variables. Since the generation and accumulation of mycotoxins during the maize cultivation season is hard to describe with a limited set of parameters, given the different maize growth periods and the influencing weather variables, involving more variables makes the model more universalized.

Additionally, BN modeling is more flexible and can predict mycotoxin contamination even when some input parameters values are missing (Liu et al., 2018). In practice, data on some of the model parameters could be inaccessible or even unavailable at all. The way BN models handle missing data ensures that the developed BN models can tolerate unstructured input data, without the need for changing or retraining the model (Wang and Yuan, 2004). This is an important asset of BN modeling for forecasting mycotoxins because it allows the model to be run already in the early maize cultivation season, when information of the entire growth period is not yet available. This asset is also the reason why the BN model could be very useful for early warning purposes. According to the results presented in Tables 2, 4, the developed BN models could deliver acceptable predictions in the early maize growth stages. Meanwhile, with more ARIs and FRIs involved in the later growth stages, PREMA and PREFUM will provide higher specificity, implying lower percentages of false negatives. The predictions for the high contaminated fields thus become more accurate when the maize cultivation season progresses, and these predictions can still provide stakeholders with the opportunity of making timely decisions on managing aflatoxin and fumonisin contamination in maize. The developed models are intended to be used by stakeholders of the maize supply chain, including farmers, buyers/collectors, and food safety authorities. Actions for managing mycotoxin contamination in maize batches that can be taken before/at harvesting are related to decisions on, e.g., keeping batches from particular fields separate, routing and processing within the maize feed and food supply chain, and risk-based monitoring.

In the current research, the two developed mycotoxin forecasting models PREMA and PREFUM were both internally and externally validated by using samples collected from the same country, both in the same time periods and in different time periods (years) as data used for model training. Model performance results showed that both PREMA and PREFUM have a better performance when using the internal validation set as compared to the external validation. This is due to the characteristics of data distribution; since the samples in the internal validation set were collected in the same time period of the samples in the training set, data on mycotoxin contamination of the internal validation set showed a similar distribution to those data from the training set. On the contrary, the samples in the external validation set were collected from different (new) years, not present in the training and internal validation set, resulting in a different mycotoxin distribution in the external validation data, partly because of variation in environmental conditions between years. It is, however, of utmost importance to perform an external model validation as well as to check the early warning performance of the model since the ultimate aim of the mycotoxin forecasting model is to use it for predicting mycotoxins during the next year’s growing season.

The methodology proposed in this study is able to overcome the obstacles of unintegrated and unbalanced dataset. Results suggest that the developed models PREMA and PREFUM can well classify the unknown samples into the correct contamination class, especially for the high contaminated samples. This is extremely valuable since, in practice, the aim is to predict and detect the high contaminated maize fields. The model performance could be further improved when more data and/or more detailed data are available for model training, such as agronomical data and more detailed weather data. Meanwhile, the current study used meteorological data from the JRC European meteorological database. It is an open source database, but it represents the meteorological grid data with a 25-km^2^ spatial resolution. Hence, the performance of the models can possibly be improved when higher-resolution meteorological data—more specific to the location of the particular maize fields—can be used in model training. Furthermore, the developed models only took meteorological factors into account. When available, agronomic data such as related to maize variety could be added into the BN model as independent nodes as well. Additionally, the developed models can be further validated by applying them into practice, and they can be extended to other European maize-growing areas.

## Data Availability Statement

The data analyzed in this study is subject to the following licenses/restrictions: Data collected in course of other project. Requests to access these datasets should be directed to FB, ferenc.bagi@polj.edu.rs.

## Author Contributions

TD, ML, and FB: sample collection and mycotoxin analyses. NL: data cleaning, model development, and manuscript writing. CL: model development and manuscript revisions. HF-K: project acquisition, manuscript writing, and revisions. All authors contributed to the article and approved the submitted version.

## Conflict of Interest

The authors declare that the research was conducted in the absence of any commercial or financial relationships that could be construed as a potential conflict of interest.

## Footnotes

1 JRC European meteorological database: https://agri4cast.jrc.ec.europa.eu/DataPortal/Index.aspx
